# Supplementary figures and images for: Design principles for inflammasome inhibition by pyrin-only-proteins
Source: eLife. 2024 Jan 22;13:e81918. doi: 10.7554/eLife.81918 (PMC10803020; doi:10.7554/eLife.81918)

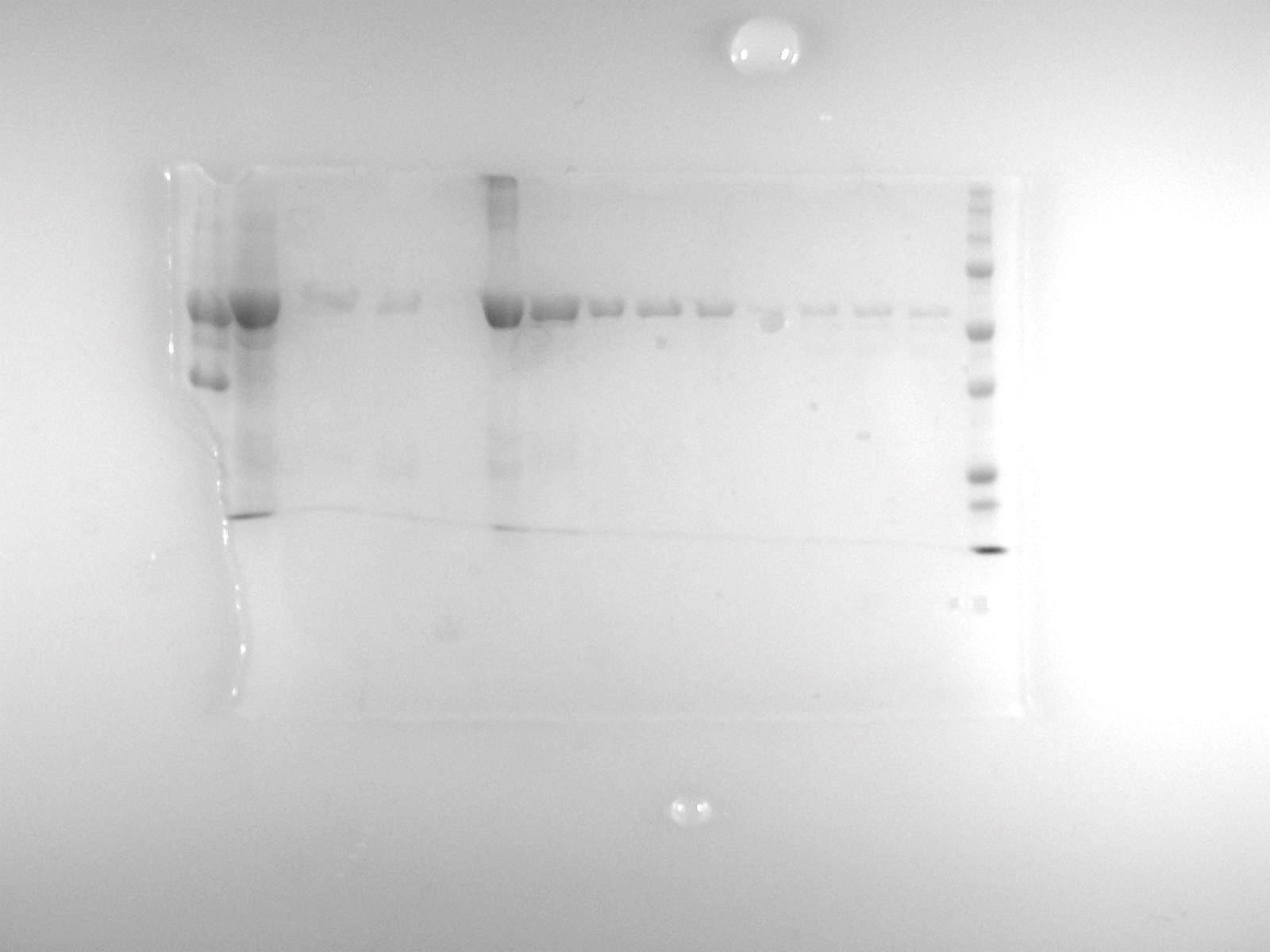

Supplement: Figure 2—source data 1. [file elife-81918-fig2-data1.zip › Figure2_SourceData1/Fig2-FS2_SourceData_Raw/POP2 gel.JPG]

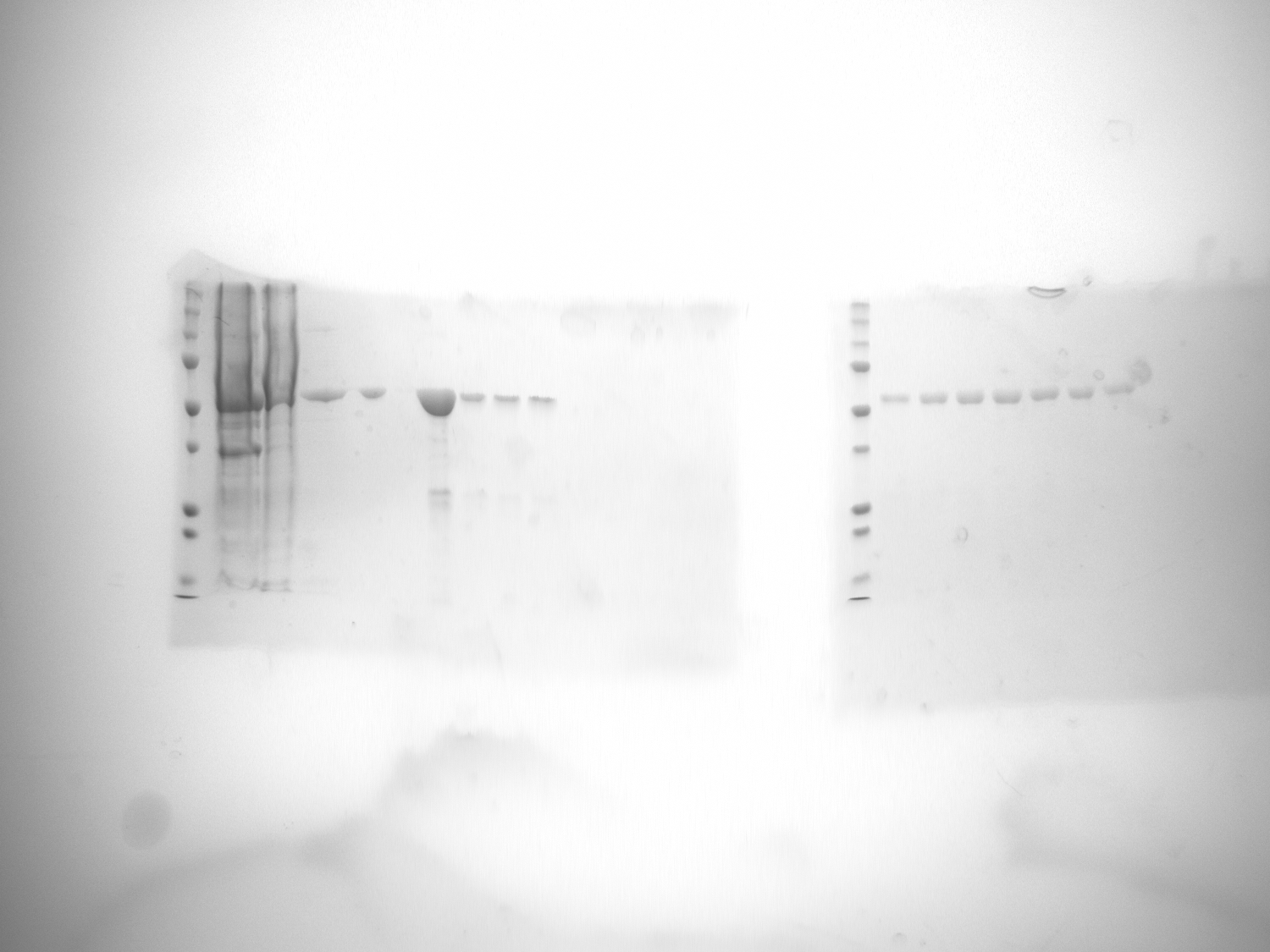

Supplement: Figure 2—source data 1. [file elife-81918-fig2-data1.zip › Figure2_SourceData1/Fig2-FS2_SourceData_Raw/POP3 gel.jpg]

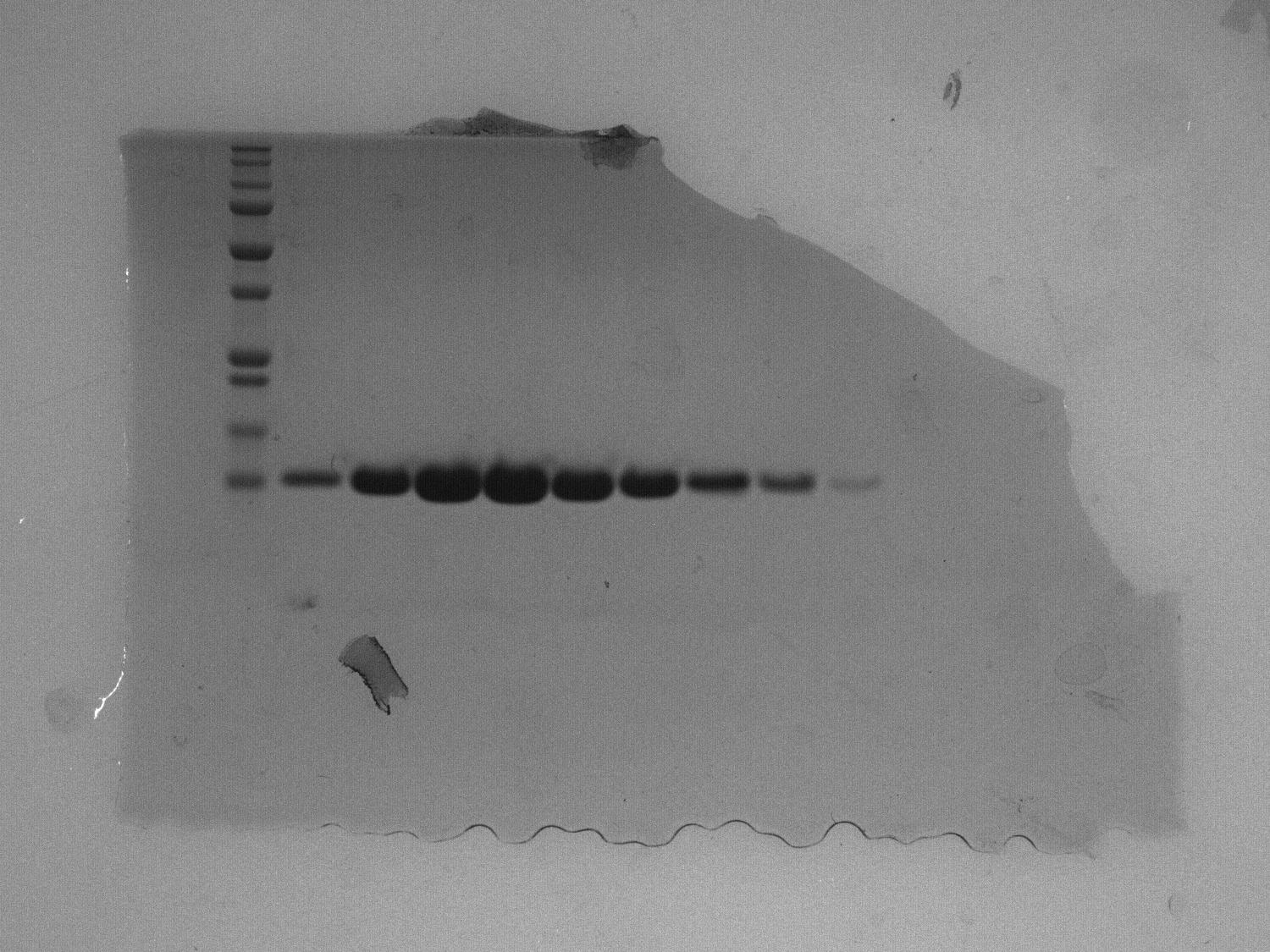

Supplement: Figure 2—source data 1. [file elife-81918-fig2-data1.zip › Figure2_SourceData1/Fig2-FS2_SourceData_Raw/POP1 gel.JPG]

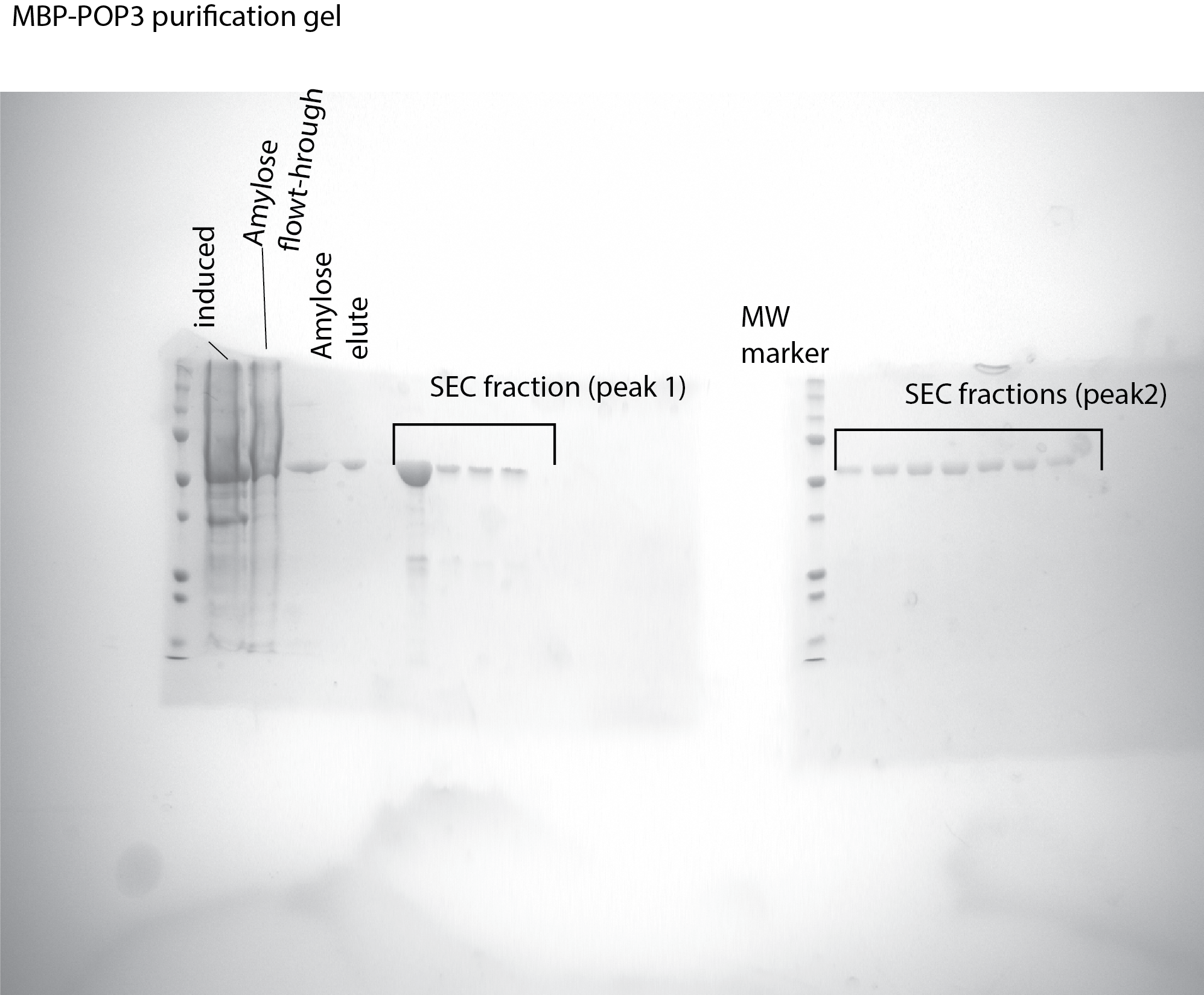

Supplement: Figure 2—source data 1. [file elife-81918-fig2-data1.zip › Figure2_SourceData1/Fig2-FS2_SourceData_Labelled/POP3source.png]

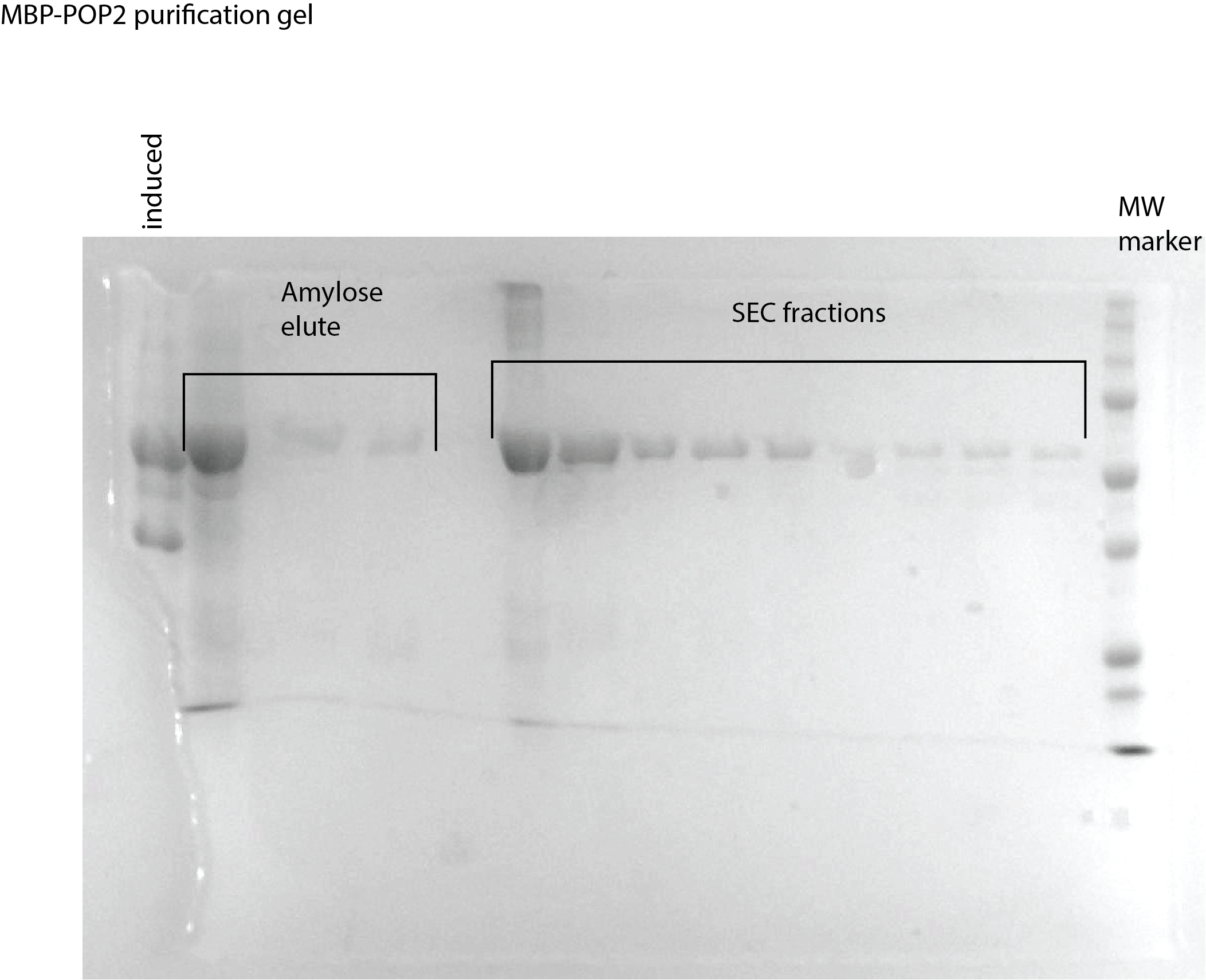

Supplement: Figure 2—source data 1. [file elife-81918-fig2-data1.zip › Figure2_SourceData1/Fig2-FS2_SourceData_Labelled/POP2source.png]

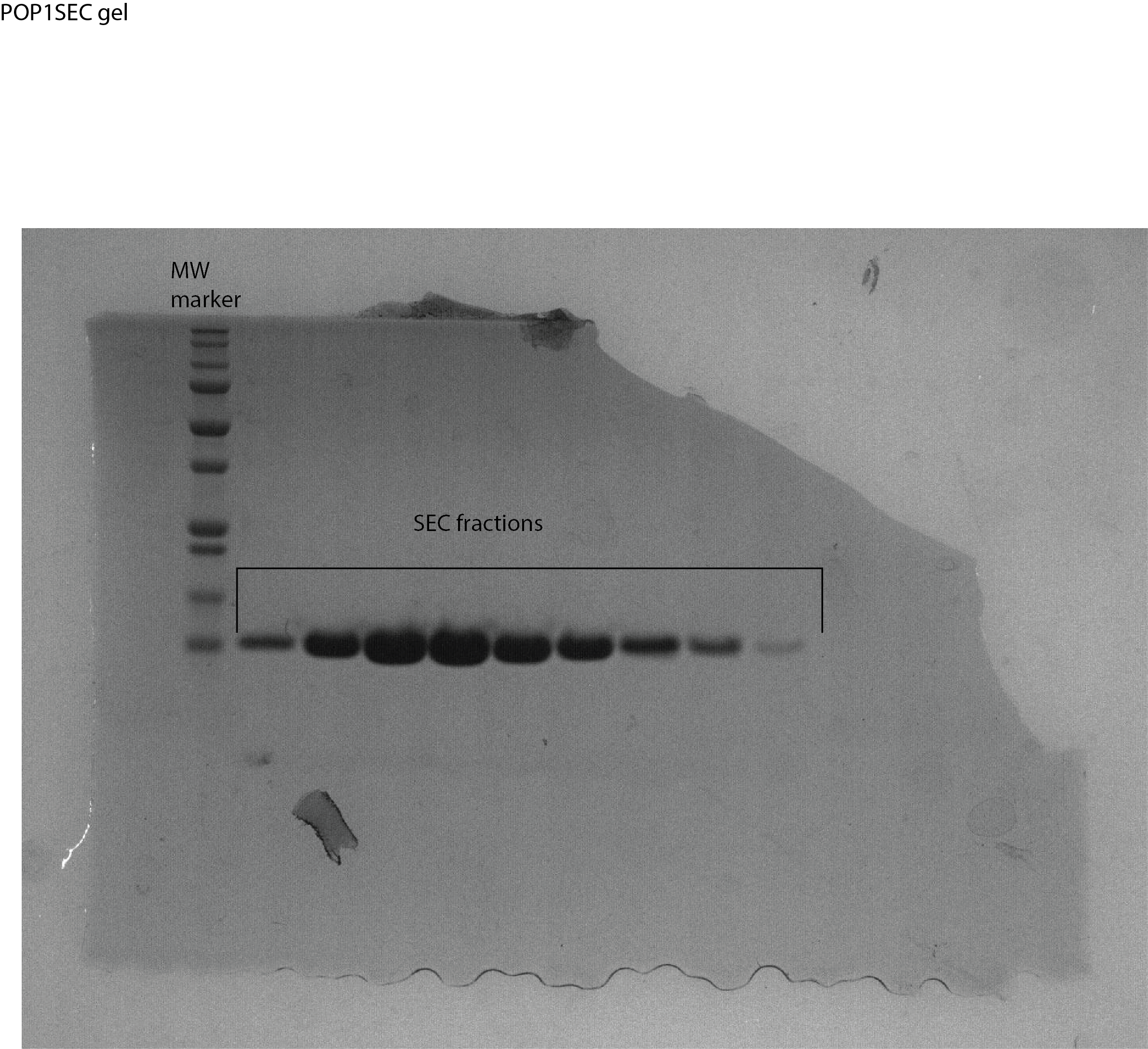

Supplement: Figure 2—source data 1. [file elife-81918-fig2-data1.zip › Figure2_SourceData1/Fig2-FS2_SourceData_Labelled/POP1source.png]
